# Investigation of serum cystatin C levels and their diagnostic value in combination with inflammatory ratios in patients with bipolar disorder

**DOI:** 10.3389/fpsyt.2025.1525091

**Published:** 2025-04-04

**Authors:** Chenjiao Zhang, Jinbao Ma, Huanqin Gao, Yanhong Luo, Junhui Feng, Yanyan Wei, Jingxu Chen

**Affiliations:** ^1^ School of Mental Health, Bengbu Medical University, Bengbu, Anhui, China; ^2^ Beijing Hui-Long-Guan Hospital, Peking University, Beijing, China; ^3^ Beijing Tong-Ren Hospital, Capital Medical University, Beijing, China; ^4^ The Sixth Department of Psychiatry, Jining Psychiatric Hospital, Jining, Shandong, China

**Keywords:** bipolar disorder, cystatin C, inflammation, biomarker, serum

## Abstract

**Background:**

It is thought that inflammation significantly contributes to the development of bipolar disorder (BD), and recent findings indicate a connection between cystatin C and immune-related inflammation. In this study, we investigated serum cystatin C levels in patients with BD and explored the relationship between cystatin C and inflammatory markers.

**Methods:**

The study involved 3,647 individuals diagnosed with BD, comprising 2,431 with BD-manic (BD-M) and 1,216 with BD-depression (BD-D), alongside 3,500 healthy controls. The analysis covered cystatin C levels and inflammatory biomarkers obtained from complete blood counts across the various groups. The Spearman correlation test was used to examine the relationship between cystatin C and inflammatory markers. Logistic regression and ROC curve analyses assessed the predictive value of these markers for disease occurrence.

**Results:**

Serum cystatin C levels were significantly elevated in BD patients, particularly those in manic episodes, compared to the healthy control group, with distinct correlation patterns with inflammatory biomarkers observed among the groups. Serum Cystatin C levels independently and positively indicated disease occurrence, showing improved diagnostic effectiveness when combined with inflammatory ratios.

**Conclusion:**

Our research indicates that cystatin C could be involved in the pathophysiological mechanisms of BD by affecting pro-inflammatory processes. Additionally, it should be emphasized that cystatin C showed considerable predictive capacity in diagnosing BD, especially when used alongside various inflammatory markers.

**Limitations:**

The cross-sectional study is limited to demonstrating associations rather than establishing causality. A thorough examination of sociodemographic factors and the severity of the disease could not be conducted.

## 1 Introduction

Bipolar disorder (BD) represents a widespread, recurring, and incapacitating psychiatric illness distinguished by fluctuating episodes of depression accompanied by either mania or hypomania, impacting about 40 million people globally (1). Individuals with BD, a significant contributor to global disability, often face increased risks of suicidal behavior, cardiovascular diseases, aggression, and legal issues, potentially impairing social functioning and reducing quality of life (2, 3). Genetic predispositions and environmental conditions contribute to BD development, but the underlying mechanisms are not fully understood (4). In clinical practice, the diagnosis of bipolar disorder (BD) relies predominantly on subjective clinical symptoms and lacks objective biomarkers. Hence, it is crucial to delve deeper into the pathophysiological processes associated with BD and to pinpoint potential biomarkers for its identification, facilitating early diagnosis and prompt treatment.

Prior studies have demonstrated that immune dysfunction and inflammation are key factors in the development of BD (5, 6). These findings underscore the critical role of comprehending immune response mechanisms and inflammatory processes in the pathogenesis of BD. Furthermore, a rising number of inflammation-related biomarkers identified through hematological assessments have been found to correlate with the onset of BD (7–9), reinforcing the connection between immune response, inflammation, and the disease. Cystatin C, a prominent cysteine protease inhibitor predominantly located in the brain, has emerged as a factor intricately linked to immune inflammation (10). Originally identified by Jorgen Clausen as a protein present in cerebrospinal fluid, cystatin C has since been recognized as a vital component in various bodily fluids (11). The CST3 gene encodes human cystatin C, found in all mammalian tissues and fluids such as cerebrospinal fluid, blood plasma, urine, semen, and saliva (12). Cystatin C is predominantly produced by astrocytes in the central nervous system, with its cerebrospinal fluid concentration being quintuple that of the bloodstream (13, 14).

While cystatin C is mainly recognized as a marker for glomerular filtration rate (GFR), its biological functions go well beyond this designated role. This protein demonstrates both antibacterial and antiviral characteristics, affects tumor metastasis, aids in bone resorption, influences the immune system, and encourages cellular proliferation and growth (15). Growing evidence indicates that cystatin C plays a direct role in various immunological disorders, with its encoding gene being regulated by inflammatory mediators like cytokines in situations of inflammation or infection (16). Cystatin C influences inflammation and immune responses by altering cysteine protease activity or through mechanisms independent of its inhibitory functions (17). Studies on serum cystatin C levels have provided significant diagnostic insights into various inflammatory diseases (18). Recent scholarly interest has increased in the relationship between serum cystatin C levels and central nervous system disorders, including Alzheimer’s, Parkinson’s, and depression (19–21). Research reveals that patients experiencing depression exhibit considerably higher cystatin C levels compared to healthy individuals, which may be linked to immunological processes (22). Another study suggested that inflammatory depression is associated with selective glomerular hypofiltration (SGHS), which are characterized by a reduced estimated glomerular filtration rate (eGFR) based on cystatin C relative to eGFR based on creatinine (23). Furthermore, the pro-inflammatory cytokine IL-6 partially mediated the relationship between SGHS and depression. Similar to depression, BD is also characterized by chronic low-grade systemic inflammation (24). Whether cystatin C levels exhibit alterations in BD is equally worth investigating. Nonetheless, previous research has not assessed serum cystatin C levels in BD patients, especially during varying episodes such as manic (BD-M) and depressive (BD-D) phases.

Prior research has shown that the neutrophil-to-lymphocyte ratio (NLR), monocyte-to-lymphocyte ratio (MLR), platelet-to-lymphocyte ratio (PLR), systemic immune-inflammation index (SII), and system inflammation response index (SIRI) are effective inflammation biomarkers, readily derived from complete blood counts (7, 8). It has also been noted that these parameters are significantly elevated in individuals with BD and exhibit strong predictive capabilities regarding the disease’s occurrence. Considering the specific association of cystatin C and the derived ratios from complete blood counts with inflammation, further investigation into their relationship is warranted. Taking into account the predictive significance of such inflammatory biomarkers, the integration of cystatin C with these ratios could yield additional insights for predicting BD.

In this study, we leveraged comprehensive clinical data to investigate cystatin C concentration differences between BD patients (primarily those in manic or depressive episodes) and healthy controls, as well as between BD-M and BD-D subgroups, while also exploring associations between cystatin C and inflammatory markers to elucidate its potential role in BD pathophysiology. We further compared the diagnostic performance of cystatin C with established inflammatory indices and evaluated their combined utility, hypothesizing that cystatin C levels would be elevated in BD patients (with differential expression across mood states), correlate with inflammatory markers, and demonstrate diagnostic accuracy comparable to conventional inflammatory biomarkers.

## 2 Subjects and methods

### 2.1 Participants

This retrospective cross-sectional study was conducted in a real-world setting at Beijing Hui-Long-Guan Hospital from January 2015 to January 2021. We examined sociodemographic and hematological data of participants obtained from the hospital’s Electronic Medical Record System (EMRS). The study focused on hospitalized patients in the acute phase of BD. Our sample comprised 3,647 BD patients, which included 2,431 BD-M patients and 1,216 BD-D patients. The diagnosis of BD as well as disease episodes were based on the criteria of International Statistical Classification of Diseases and Related Health Problems, 10th Revision (ICD-10). Participants were selected if they met these criteria: (a) a verified ICD-10 diagnosis of BD by two independent senior psychiatrists (BD-M: F31.0, F31.1, F31.2; BD-D: F31.3, F31.4, F31.5): (b) aged 18 to 65 years; and (c) availability of hematological data for Cystatin C, platelets, neutrophils, lymphocytes, and monocytes. The exclusion criteria encompassed: (a) the presence of comorbid psychiatric disorders;(b) significant infections, autoimmune disorders, malignant tumors, renal illnesses, liver diseases, diabetes, obesity, or pregnancy; and (c) prior use of nonsteroidal anti-inflammatory medications, steroids, or antibiotics within a month. In a parallel effort to establish a control group, we included 3,500 healthy individuals who were retrospectively selected from the EMRS of the Health Examination Center at Beijing Tong-Ren Hospital during the same study period. The healthy control group was composed of individuals who went to the hospital for routine health examinations. They had no current or past diagnoses of any mental disorders based on ICD-10 criteria, were between 18 and 65 years old, and met the same exclusion criteria as the patients.

### 2.2 Data collection and calculation

The study involved the collection of both fundamental demographic characteristics, such as age and sex, as well as various hematological parameters. These parameters included levels of Cystatin C, uric acid, creatinine, urea, and the counts of platelets, neutrophils, lymphocytes, and monocytes. Data was sourced from EMRS, ensuring accuracy and reliability in the demographic and hematological information gathered. Fasting venous blood samples were collected from participants between 7 and 9 a.m. These samples were then subjected to analysis by professional laboratory technicians, who conducted routine blood tests alongside a series of biochemical assessments to generate comprehensive results. In analyzing the inflammatory responses in participants, particular calculations for various inflammatory ratios were employed: NLR = neutrophil/lymphocyte; MLR = monocyte/lymphocyte; PLR = platelet/lymphocyte; SII = platelet × neutrophil/lymphocyte; SIRI = monocyte × neutrophil/lymphocyte.

### 2.3 Statistical analysis

All statistical analyses were performed using SPSS version 25.0. Continuous variables are expressed as mean ± standard deviation, and categorical variables are represented as counts (percentages). The chi-square (*χ²*) test was utilized for the analysis of categorical data. The Kolmogorov-Smirnov test was used to assess the normal distribution of the study variables. The Q-Q plots and histograms suggest that the variables exhibit an approximate normal distribution. Given the large sample size in this study, continuous variables were compared using an independent t-test for two groups or a one-way ANOVA for three groups. An analysis of covariance (ANCOVA) within the General Linear Model framework was conducted to assess differences in biochemical test results and complete blood count across various categories. For each laboratory measurement, ANCOVA was performed with the laboratory data serving as the dependent variable, fixed factors comprised of the diagnostic groups, and covariates including age and sex. *Post-hoc* analysis, utilizing the Bonferroni correction, was carried out to examine inter-group differences. Spearman correlation analysis was used to examine the relationships between cystatin C, age, and inflammatory biomarkers. A logistic regression analysis was performed to evaluate the predictive value of cystatin C and inflammatory ratios in relation to the occurrence of BD, encompassing both manic and depressive episodes. ROC curves were utilized to assess the predictive accuracy of individual biomarkers and combined cystatin C models with various ratios for detecting BD across different episodes. Statistical significance was defined as *P* < 0.05.

## 3 Result

### 3.1 Comparison of variables between BD and HC groups

**Figure 1:**
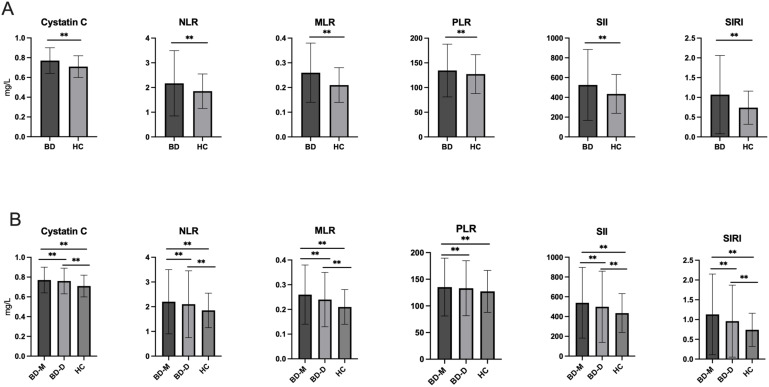
**(A)** Comparison of cystatin C, NLR, MLR, PLR, SII and SIRI between BD and HC; **(B)** Comparison of cystatin C NLR, MLR, PLR, SII and SIRI among BD-M, BD-D and HC groups. BD, Bipolar disorder; BD-M, bipolar disorder in manic episode; BD-D, bipolar disorder in depressive episode; HC, healthy controls; NLR, neutrophil-to-lymphocyte ratio; MLR, monocyte-to-lymphocyte ratio; PLR, platelet-to-lymphocyte ratio; SII, systemic immune-inflammation index; SIRI, system inflammation response index. ^**^ *P* < 0.001.

There were no significant differences of age or sex between the BD and HC groups (*P* > 0.05). Importantly, individuals with BD showed significantly elevated cystatin C levels when compared to the HCs (*P <*0.001). The study found elevated creatinine levels and decreased uric acid and urea levels among the other variables analyzed (*P* < 0.001 for all). Besides, BD patients exhibited significantly elevated levels of platelets, neutrophils, monocytes, NLR, MLR, PLR, SII, and SIRI (*P* < 0.001 for all). The results were shown in **Table 1** and **Figure 1A**.

**Table 1:** Comparison of variables between BD and HC groups.

Variables	BD (n=3,647)	HC (n=3,500)	*t/χ²*	*P*
Sex (male (n%))	1850 (50.7%)	1758 (50.2%)	0.177	0.674
Age (years)	39.92 ± 12.60	40.23 ± 11.14	1.115	0.265
Cystatin C (mg/L)	0.77 ± 0.13	0.71 ± 0.11	-20.175	0.000
Uric acid (μmol/L)	325.21 ± 95.22	342.20 ± 90.35	7.550	0.000
Creatinine (μmol/L)	71.56 ± 11.46	70.07 ± 13.51	-4.900	0.000
Urea (mmol/L)	4.58 ± 1.39	4.80 ± 1.20	7.370	0.000
Platelet (10³/µl)	241.02 ± 59.37	234.56 ± 49.37	-5.014	0.000
Neutrophil (10³/µl)	3.85 ± 1.69	3.43 ± 1.09	-12.416	0.000
Lymphocyte (10³/µl)	1.96 ± 0.65	1.95 ± 0.52	-1.165	0.244
Monocyte (10³/µl)	0.47 ± 0.18	0.39 ± 0.12	-20.698	0.000
NLR	2.17 ± 1.32	1.85 ± 0.70	-12.890	0.000
MLR	0.26 ± 0.12	0.21 ± 0.07	-20.175	0.000
PLR	134.56 ± 53.40	127.28 ± 39.29	-6.581	0.000
SII	525.62 ± 358.69	434.88 ± 196.49	-13.334	0.000
SIRI	1.07 ± 0.99	0.74 ± 0.42	-18.693	0.000

The independent t-test were used for comparison of continuous data, and *χ²* test was used for categorical data.

BD, Bipolar disorder; HC, Healthy controls; NLR, neutrophil-to-lymphocyte ratio; MLR, monocyte-to-lymphocyte ratio; PLR, platelet-to-lymphocyte ratio; SII, systemic immune-inflammation index; SIRI, system inflammation response index.

### 3.2 Comparison of variables among BD-M, BD-D, HC groups

**Table 2** categorizes participants in the BD group into two subgroups: BD-M and BD-D. Chi-square analysis indicated no significant sex differences among the BD-D, BD-M, and HC groups (*P* > 0.05). ANOVA and *post hoc* tests revealed that the BD-M group (39.57 ± 12.49) was significantly younger than the BD-D group (40.62 ± 12.75) (*P* = 0.036). After adjusting for age and sex, significant differences were observed in cystatin C, uric acid, creatinine, urea, platelets, neutrophils, monocytes, NLR, MLR, PLR, SII, and SIRI levels among the three groups (*P* < 0.001 for all).The BD-M group showed significantly higher levels of cystatin C, creatinine, platelets, neutrophils, monocytes, NLR, MLR, PLR, SII, and SIRI (*P* < 0.001) compared to the HC group, along with reduced levels of uric acid and urea. The BD-D group exhibited higher levels of cystatin C, creatinine, neutrophils, monocytes, NLR, MLR, PLR, SII, and SIRI, and lower levels of uric acid and urea compared to the HC group (*P* < 0.001). Comparisons revealed that the BD-M group exhibited elevated levels of cystatin C, uric acid, platelets, neutrophils, monocytes, NLR, MLR, PLR, SII, and SIRI, but a reduced level of creatinine compared to the BD-D group (*P* < 0.001). **Figure 1B** illustrates the differences in cystatin C, NLR, MLR, PLR, SII, and SIRI across all groups.

**Table 2:** Comparison of variables among BD-M, BD-D, HC groups.

Variables	BD-M (n=2431)	BD-D (n=1216)	HC (n=3500)	*F/χ²*	*P*
Sex (male (n%))	1243 (51.1%)	607 (49.9%)	1758 (50.2%)	0.655	0.721
Age (years)	39.57 ± 12.49b^*^	40.62 ± 12.75	40.23 ± 11.14	3.765	0.023
Cystatin C (mg/L)	0.77 ± 0.13 a^**^b^**^	0.76 ± 0.13 a^**^	0.71 ± 0.11	217.159	0.000
Uric acid (μmol/L)	331.67 ± 95.91a^**^b^**^	312.32 ± 92.53 a^**^	342.20 ± 90.35	47.713	0.000
Creatinine (μmol/L)	71.15 ± 11.44 a^**^b^**^	72.40 ± 11.47 a^**^	70.07 ± 13.51	16.840	0.000
Urea (mmol/L)	4.55 ± 1.39 a^**^	4.61 ± 1.36 a^**^	4.80 ± 1.20	29.842	0.000
Platelet (10³/µl)	243.69 ± 59.35 a^**^b^**^	235.69 ± 59.07	234.56 ± 49.37	21.195	0.000
Neutrophil (10³/µl)	3.95 ± 1.73 a^**^b^**^	3.64 ± 1.59 a^**^	3.43 ± 1.09	95.752	0.000
Lymphocyte (10³/µl)	1.98 ± 0.66	1.94 ± 0.63	1.95 ± 0.52	3.246	0.039
Monocyte (10³/µl)	0.48 ± 0.18 a^**^b^**^	0.44 ± 0.15 a^**^	0.39 ± 0.12	253.280	0.000
NLR	2.20 ± 1.30 a^**^b^**^	2.10 ± 1.35 a^**^	1.85 ± 0.70	85.580	0.000
MLR	0.26 ± 0.12 a^**^b^**^	0.24 ± 0.11 a^**^	0.21 ± 0.07	218.209	0.000
PLR	135.27 ± 54.27 a^**^	133.14 ± 51.60 a^**^	127.28 ± 39.29	22.224	0.000
SII	539.10 ± 357.31 a^**^b^**^	498.66 ± 360.05 a^**^	434.88 ± 196.49	94.977	0.000
SIRI	1.13 ± 1.02 a^**^b^**^	0.96 ± 0.91 a^**^	0.74 ± 0.42	189.772	0.000

The *χ²* test was used for categorical data, ANOVA was used for comparison of age, ANCOVA was applied for comparison of laboratory variables.

BD-M, bipolar disorder in manic episode; BD-D, bipolar disorder in depressive episode; HC, healthy controls; NLR, neutrophil-to-lymphocyte ratio; MLR, monocyte-to-lymphocyte ratio; PLR, platelet-to-lymphocyte ratio; SII, systemic immune-inflammation index; SIRI, system inflammation response index.

^*^ *P* < 0.05; ^**^ *P* < 0.001.

a, Compared with the HC group. b, Compared with the BD-D group.

### 3.3 Comparison of cystatin C among all groups with different sex or psychotic symptoms

**Figure 2:**
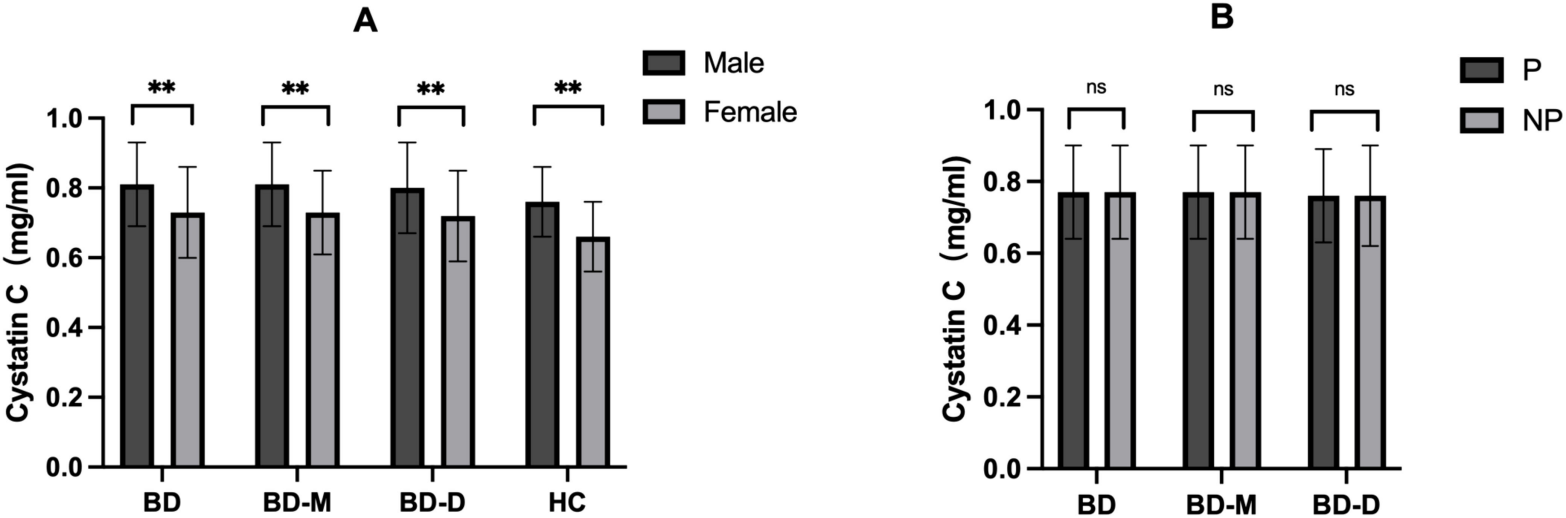
**(A)** Comparison of cystatin C among groups with different sex; **(B)** Comparison of cystatin C among groups with or without psychotic symptoms. BD, Bipolar disorder; BD-M, bipolar disorder in manic episode; BD-D, bipolar disorder in depressive episode; HC, healthy controls; P, patients with psychotic symptoms; NP, patients without psychotic symptoms. ^**^ *P* < 0.001; ns no significance.

We examined cystatin C levels in different groups, considering different clinical presentations and sex. According to ICD-10, we categorized patients into two groups based on the presence of psychotic symptoms: P (psychotic, ICD-10: F31.2, F31.5), or NP (non-psychotic, ICD-10: F31.0, F31.1, F31.3, F31.4). **Table 3** and **Figure 2** indicate no significant differences in cystatin C levels between patients with and without psychotic symptoms in the BD, BD-M, and BD-D groups (*P* > 0.05 for all). Nonetheless, both the BD cohort and the healthy control group displayed notably elevated cystatin C levels in male participants compared to their female counterparts (*P <*0.001 for all).

**Table 3:** Comparison of cystatin C among all groups with different sex or psychotic symptoms.

Groups		Cystatin C (mg/L)	*t*	*P*
BD				
	Male (n = 1850)	0.81 ± 0.12	19.408	0.000
	Female (n = 1917)	0.73 ± 0.13		
	P (n = 1526)	0.77 ± 0.13	-0.691	0.489
	NP (n = 1721)	0.77 ± 0.13		
BD-M				
	Male (n = 1243)	0.81 ± 0.12	15.906	0.000
	Female (n = 1188)	0.73 ± 0.12		
	P (n =1003)	0.77 ± 0.13	-0.706	0.474
	NP (n =1428)	0.77 ± 0.13		
BD-D				
	Male (n = 607)	0.80 ± 0.13	10.495	0.000
	Female (n = 609)	0.72 ± 0.13		
	P (n = 523)	0.76 ± 0.13	-0.275	0.784
	NP (n = 2121)	0.76 ± 0.14		
HC				
	Male (n =1758)	0.76 ± 0.10	29.629	0.000
	Female (n =1742)	0.66 ± 0.10		

The independent t-test was used for comparison of cystatin C serum concentrations.

BD, Bipolar disorder; BD-M, bipolar disorder in manic episode; BD-D, bipolar disorder in depressive episode; HC, healthy controls; P, psychotic; NP, non-psychotic.

### 3.4 Correlation coefficients between cystatin C and other variables among all groups

**Table 4** demonstrates a positive correlation between age and Cystatin C levels across all study groups (*P* < 0.001). In the HC group, cystatin C levels positively correlated with neutrophil, lymphocyte, and monocyte counts, as well as NLR, MLR, SII, and SIRI (*P* < 0.05). In contrast, a negative correlation was found between cystatin C levels and PLR (*P <*0.001). In the BD and BD-M groups, cystatin C levels showed positive correlations with neutrophils, lymphocytes, monocytes, MLR, and SIRI, while displaying negative correlations with platelets, PLR, and SII. In the BD-D group, cystatin C showed a positive correlation with lymphocyte and monocyte counts, and a negative correlation with platelet count, PLR, and SII.

**Table 4:** Correlation coefficients between cystatin C and other variables.

Variables	Cys C			
	HC	BD	BD-M	BD-D
age	0.283^**^	0.197^**^	0.212^**^	0.175^**^
Neutrophils	0.176^**^	0.044^**^	0.042^**^	0.033
Lymphocytes	0.145^**^	0.071^**^	0.067^**^	0.076^**^
Monocytes	0.265^**^	0.130^**^	0.154^**^	0.067^*^
Platelet	0.005	-0.092^**^	-0.107^**^	-0.072^*^
NLR	0.049^**^	-0.010	-0.007	-0.030
MLR	0.138^**^	0.062^**^	0.085^**^	0.001
PLR	-0.124^**^	-0.122^**^	-0.125^**^	-0.118^**^
SII	0.041^*^	-0.045^**^	-0.048^*^	-0.056^*^
SIRI	0.196^**^	0.067^**^	0.081^**^	0.025

Results given as Spearman correlation coefficient.

BD, Bipolar disorder; BD-M, bipolar disorder in manic episode; BD-D, bipolar disorder in depressive episode; HC, healthy controls; NLR, neutrophil-to-lymphocyte ratio; MLR, monocyte-to-lymphocyte ratio; PLR, platelet-to-lymphocyte ratio; SII, systemic immune-inflammation index; SIRI, system inflammation response index.

^*^ *P* < 0.05; ^**^ *P* < 0.001.

### 3.5 The prognostic significance of cystatin C and inflammatory markers for BD, BD-M, and BD-D

**Figure 3:**
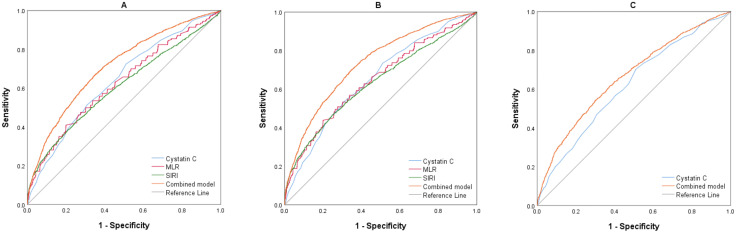
**(A)** ROC curves of the parameters for BD (BD vs. HC). **(B)** ROC curves of the parameters for BD-M (BD-M vs. HC). **(C)** ROC curves of the parameters for BD-D (BD-D vs. HC). BD, Bipolar disorder; BD-M, bipolar disorder in manic episode; BD-D, bipolar disorder in depressive episode; MLR, monocyte/lymphocyte ratio; SIRI, system inflammation response index.

**Table 5** demonstrates that the binary logistic regression model for BD included age, sex, cystatin C, NLR, MLR, PLR, and SIRI, each independently linked to BD development. Moreover, sex, cystatin C, MLR, PLR, and SIRI exhibited positive correlations with BD, whereas age and NLR displayed negative associations. Aligning with the BD model, sex, cystatin C, MLR, PLR, and SIRI emerged as positive indicators for BD-M, while age and NLR were again inversely related to BD-M. In the BD-D subgroup, sex, cystatin C, MLR, and SIRI showed positive correlations with BD-D, whereas age was negatively associated. ROC curves were utilized to assess the diagnostic value of individual indicators like cystatin C, NLR, MLR, PLR, SII, and SIRI, along with combined models from previous logistic regression analyses. The findings are summarized in **Tables 6**–**9** and **Figure 3**. In classifying BD, cystatin C had higher AUC than any other indicators, however, only the combined model achieved an AUC above 0.7. The results for the BD-M group were similar to those of the BD group. In the BD-D category, even the combined model showed the AUC value that did not exceed 0.7. We conducted further ROC analysis to differentiate between BD-M and BD-D; however, the AUC values for all indicators remained below 0.6.

**Table 5:** Results of Binary logistic regression analysis.

Variables	BD			BD-M			BD-D		
	OR	95%CI	*P*	OR	95%CI	*P*	OR	95%CI	*P*
Sex	1.828	1.633-2.047	0.000	1.943	1.708-2.211	0.000	1.679	1.444-1.953	0.000
Age	0.985	0.981-0.99	0.000	0.981	0.976-0.986	0.000	0.990	0.984-0.996	0.001
Cystatin C	190.931	113.99-319.805	0.000	276.051	153.599-496.127	0.000	77.350	40.111-149.163	0.000
NLR	0.673	0.594-0.763	0.000	0.616	0.533-0.711	0.000	–	–	–
MLR	12.259	3.767-39.891	0.000	8.978	2.386-33.799	0.001	19.780	5.164-75.768	0.000
PLR	1.003	1.001-1.004	0.001	1.004	1.002-1.006	0.000	–	–	–
SII	–	–	–	–	–	–	–	–	–
SIRI	2.728	2.12-3.51	0.000	3.561	2.673-4.744	0.000	1.258	1.036-1.526	0.020

BD, Bipolar disorder; BD-M, bipolar disorder in manic episode; BD-D, bipolar disorder in depressive episode; NLR, neutrophil-to-lymphocyte ratio; MLR, monocyte-to-lymphocyte ratio; PLR, platelet-to-lymphocyte ratio; SII, systemic immune-inflammation index; SIRI, system inflammation response index.

**Table 6:** Results of ROC curves of the parameters for BD vs. HC.

Variables	AUC	95%*CI*	*P*	Cut-off	Sensitivity	Specificity
Cystatin C	0.640	0.628-0.653	0.000	0.70	0.724	0.490
NLR	0.550	0.536-0.563	0.000	2.43	0.291	0.835
MLR	0.627	0.614-0.640	0.000	0.25	0.410	0.800
PLR	0.524	0.511-0.537	0.001	148.60	0.319	0.763
SII	0.555	0.542-0.549	0.000	569.93	0.321	0.817
SIRI	0.608	0.595-0.621	0.000	0.90	0.426	0.750
Combined model	0.712	0.700-0.724	0.000	–	0.667	0.635

BD, Bipolar disorder; HC, healthy controls; NLR, neutrophil-to-lymphocyte ratio; MLR, monocyte-to-lymphocyte ratio; PLR, platelet-to-lymphocyte ratio; SII, systemic immune-inflammation index; SIRI, system inflammation response index.

**Table 7:** Results of ROC curves of the parameters for BD-M vs. HC.

Variables	AUC	95%*CI*	*P*	Cut-off	Sensitivity	Specificity
Cystatin C	0.652	0.637-0.666	0.000	0.76	0.534	0.693
NLR	0.565	0.549-0.580	0.000	2.33	0.334	0.806
MLR	0.647	0.633-0.662	0.000	0.25	0.439	0.800
PLR	0.528	0.513-0.543	0.000	148.60	0.322	0.763
SII	0.575	0.559-0.590	0.000	588.81	0.339	0.815
SIRI	0.634	0.619-0.648	0.000	0.91	0.466	0.753
Combined model	0.735	0.723-0.748	0.000	–	0.699	0.655

BD-M, bipolar disorder in manic episode; HC, healthy controls; NLR, neutrophil-to-lymphocyte ratio; MLR, monocyte-to-lymphocyte ratio; PLR, platelet-to-lymphocyte ratio; SII, systemic immune-inflammation index; SIRI, system inflammation response index.

**Table 8:** Results of ROC curves of the parameters for BD-D vs. HC.

Variables	AUC	95%*CI*	*P*	Cut-off	Sensitivity	Specificity
Cystatin C	0.618	0.600-0.636	0.000	0.70	0.703	0.490
NLR	0.519	0.499-0.540	0.043	2.46	0.269	0.842
MLR	0.586	0.567-0.605	0.000	0.25	0.352	0.800
PLR	0.516	0.496-0.536	0.094	152.05	0.295	0.786
SII	0.517	0.497-0.583	0.076	578.64	0.283	0.826
SIRI	0.556	0.536-0.576	0.000	1.11	0.251	0.863
Combined model	0.663	0.645-0.681	0.000		0.543	0.700

BD-D, bipolar disorder in depressive episode; HC, healthy controls; NLR, neutrophil-to-lymphocyte ratio; MLR, monocyte-to-lymphocyte ratio; PLR, platelet-to-lymphocyte ratio; SII, systemic immune-inflammation index; SIRI, system inflammation response index.

**Table 9:** Results of ROC curves of the parameters for BD-M vs. BD-D.

Variables	AUC	95%*CI*	*P*	Cut-off	Sensitivity	Specificity
Cystatin C	0.535	0.515-0.555	0.000	0.78	0.474	0.606
NLR	0.538	0.518-0.558	0.000	2.04	0.443	0.632
MLR	0.561	0.541-0.581	0.000	0.23	0.548	0.562
PLR	0.510	0.490-0.530	0.307	117.67	0.573	0.457
SII	0.550	0.530-0.569	0.000	383.30	0.620	0.470
SIRI	0.572	0.552-0.591	0.000	0.83	0.526	0.609
Combined model	0.576	0.556-0.596	0.000	–	0.485	0.635

BD-M, bipolar disorder in manic episode; BD-D, bipolar disorder in depressive episode; NLR, neutrophil-to-lymphocyte ratio; MLR, monocyte-to-lymphocyte ratio; PLR, platelet-to-lymphocyte ratio; SII, systemic immune-inflammation index; SIRI, system inflammation response index.

## 4 Discussion

This retrospective analysis found significantly elevated serum cystatin C levels in individuals with BD compared to healthy controls. While such findings might suggest renal dysfunction within the BD cohort, the uric acid, urea, and creatinine results provide contradictory evidence. Specifically, the BD group displayed notably elevated creatinine levels paired with reduced uric acid and urea levels, hinting at the potential influence of other mechanisms on cystatin C levels. Recent studies have explored the link between high serum cystatin C levels and psychiatric disorders, particularly depression. A cohort study involving 1,440 Chinese seniors over 60 identified a detrimental association between elevated serum cystatin C levels and heightened depression risk (25). An online cross-sectional study of 159 major depressive disorder (MDD) patients revealed a significant correlation between serum cystatin C levels and depressive symptom severity (22). Cystatin C has various biological functions potentially influencing depression development. Although BD and MDD are categorized as distinct clinical entities, they share etiological connections and clinical characteristics, including depressive symptoms (26), implying that the mechanisms through which cystatin C affects physiological and pathological states in MDD might also manifest in BD. BD, like MDD, is associated with systemic low-grade inflammation (27), marked by increased levels of inflammatory cytokines including IL-6, TNF-α, IL-1RA, and sIL-2R (28). Cystatin C, commonly used as a marker for glomerular filtration rate in clinical settings, is increasingly recognized for its role in immune responses to external and internal antigens in pathological conditions. This underscores cystatin C’s wider clinical significance, extending beyond mere kidney function assessment. The elevated serum levels of cystatin C observed in BD in our study may be attributed to its association with immune inflammation and modulation by cytokines, similar to findings in MDD reported in previous research.

This study found higher cystatin C levels in BD-M patients compared to those with BD-D. The clinical manifestations of the two episodes are distinct: the manic episode typically features elevated mood and increased energy or activity, while the depressive episode is marked by sadness and lack of pleasure (29). A study encompassing 8,332 BD patients revealed that elevated levels of CRP during manic episodes persisted even after adjustments for confounding variables, including age, sex, BMI, psychotic symptoms, and age at onset (30). This finding suggests an independent association between inflammatory changes and mood episodes. In comparison to depressive episodes, manic episodes might correlate with a more pronounced inflammatory response, which could influence cystatin C levels.

BD patients with psychotic symptoms have poorer outcomes than non-psychotic individuals, as indicated by higher hospitalization rates and longer stays (31). A study of 665 inpatients analyzed biochemical variables in BD patients with and without psychotic features. Findings revealed that patients with psychotic symptoms exhibited a higher NLR and lower total cholesterol and triglyceride levels, suggesting increased inflammation and reduced metabolic alterations (32). A recent study investigated associations between peripheral blood biomarkers (including cystatin C) and symptom severity across MDD, BD, and schizophrenia. Notably, cognitive-related symptoms such as auditory hallucinations were significantly correlated with elevated cystatin C levels (33). However, our study found no significant differences in cystatin C levels between patients with and without psychotic symptoms in both BD-M and BD-D groups. Since our study was cross-sectional, further research is needed to evaluate the possible link between psychotic symptoms and cystatin C levels. This study investigated the effects of sex and age on cystatin C levels in the BD, BD-M, BD-D, and HC groups. Our study found that males had higher cystatin C levels than females, and cystatin C levels positively correlated with age across all groups. This suggests that cystatin C concentrations may rise as age increases. These findings align with several prospective cross-sectional cohort studies in healthy populations (34, 35) and our study further corroborates these results in patients with BD.

Recently, inflammation ratios derived from complete blood counts have gained attention as superior biomarkers for indicating inflammation states. NLR, MLR, PLR, SII, and SIRI are derived from various combinations of neutrophils, lymphocytes, monocytes, and platelets. Neutrophils, lymphocytes, and monocytes are distinct white blood cell types that play unique roles in the innate and adaptive immune systems, contributing to defense against infections and immune-related diseases (36). Platelets, produced in the bone marrow, are crucial for hemostasis, wound healing, and angiogenesis; moreover, their role in inflammation across various diseases is supported by a substantial body of evidence (37). NLR, MLR, PLR, SII, and SIRI have been extensively studied in immune-inflammatory conditions like infectious inflammation and tumors (38–41), as well as in mental health disorders such as affective disorders and schizophrenia (7, 9, 42, 43). Our study revealed that all five ratios were elevated in BD patients, irrespective of manic or depressive episodes, compared to healthy controls. Furthermore, NLR, MLR, SII, and SIRI were significantly higher during manic episodes than during depressive episodes, highlighting the increased level of inflammation in manic episode, as demonstrated in previous research. The relationship between cystatin C and inflammatory markers differed among diagnostic groups and healthy controls. Cystatin C exhibited a weak yet significant correlation with all inflammatory ratios; however, this relationship may be influenced by different disease stages. A significant correlation between cystatin C and the NLR was observed exclusively in the HC group. Moreover, the HC group displayed higher correlation coefficients for MLR and SIRI in comparison to the diagnostic groups. This difference might be due to a more robust correlation involving cystatin C with neutrophils, lymphocytes, and monocytes within the HC group, along with the notable negative correlation between cystatin C and platelets that was found only in the disease groups. We propose that different stages of BD may involve distinct interactions affecting the relationship between cystatin C and immune cells such as neutrophils, lymphocytes, monocytes, and platelets. Nonetheless, as this study is cross-sectional in nature, additional research is necessary to investigate the pathophysiological connections linking cystatin C with these inflammatory biomarkers in BD.

In clinical practice, the diagnosis of BD largely depends on clinical features such as affective temperaments and negative clinical outcomes (44), which may not be sufficiently effective for the early detection of the disease due to its varied and confusing symptoms. Consequently, efforts have been directed toward identifying objective and reliable biological predictors to facilitate timely and efficient diagnosis (45). In recent years, a substantial body of research has investigated inflammation and oxidative stress-related peripheral blood biomarkers derived from complete blood counts or biochemical test data in BD (46, 47). Research indicates that NLR, MLR, PLR, SII, and SIRI are effective predictors of BD occurrence. However, the efficacy of these indicators is not entirely satisfactory, indicating that the search for more useful biomarkers remains worthwhile in the future. Meanwhile, cystatin C has been noted in some psychiatric disorders, and elevated serum cystatin C levels suggest potential diagnostic capabilities in these diseases (19, 21). In our research, we observed increased concentrations of cystatin C in patients with BD when compared to healthy individuals, highlighting its considerable potential in forecasting various stages of BD. **Table 5**’s binary logistic regression analysis identified serum cystatin C levels as independent and positive predictors of disease occurrence, particularly in the BD-M group. We performed receiver operating characteristic curve analyses to further investigate the diagnostic effectiveness of specific indicators, including cystatin C, NLR, MLR, PLR, SII, and SIRI, as well as combined models derived from previous logistic regression results. Cystatin C demonstrated the highest diagnostic effectiveness among the parameters evaluated, indicating its strong diagnostic potential in diagnosing BD. The logistic regression combined models demonstrated superior diagnostic effectiveness. The results indicate that cystatin C may act as a significant biomarker for diagnosing BD, especially when combined with other inflammatory markers. Moving forward, our efforts will focus on identifying more effective biomarkers and improving the integration of these indicators.

Our current study has various strengths and limitations. This study provides valuable real-world insights, enhancing knowledge in the field and underscoring its clinical importance. To the best of our understanding, this is the first large-scale study to analyze cystatin C levels across different episodes of BD and evaluate its diagnostic potential, both alone and in combination with other inflammatory ratios. This study represents the most comprehensive analysis of serum cystatin C levels and their association with peripheral inflammation ratios in BD to date. Nonetheless, it is crucial to recognize several limitations. Primarily, since this is a retrospective analysis, the severity of symptoms, illness duration, and frequency of episodes could not be assessed through structured psychiatric evaluations. Consequently, this study did not explore the relationship between cystatin C levels and disease severity. We did not perform a more detailed grouping based on ICD-10 criteria, and therefore cannot investigate changes in indicators across additional disease stages of BD. Due to feasibility constraints, we were unable to collect additional sociodemographic variables, including smoking habits, nutritional practices, body mass index, and social status. These variables may affect cystatin C levels and inflammation ratios. Our study did not consider treatment effects of medications, which may influence the levels of these indicators. Lastly, given that this is a cross-sectional analysis, it can only illustrate associations rather than establish causality. In the future, we intend to carry out more comprehensive research to explore the relationships between cystatin C and inflammatory biomarkers in more detailed BD subgroups over an extended period.

## 5 Conclusion

In this research, we noted increased serum concentrations of cystatin C among patients with BD, particularly during manic episodes. Our results further revealed a correlation between cystatin C levels and various immune cells, such as neutrophils, lymphocytes, monocytes, and platelets, along with derived biomarkers like the NLR, MLR, PLR, SII, and SIRI, indicating a relationship between cystatin C and inflammation. Additionally, our findings suggested that cystatin C might act as an independent risk factor for BD, including both depressive and manic phases, in comparison to healthy controls. We discovered that cystatin C exhibited superior diagnostic efficacy relative to other inflammatory indices, and an integrated model incorporating these factors displayed enhanced diagnostic capacity. In summary, our findings highlight the importance of cystatin C in BD, proposing its potential role in facilitating the diagnosis of this disorder in the future. We advocate for more research on cystatin C, which could yield deeper insights into its inflammatory roles and contribute to our comprehension of the pathophysiology of BD and various psychiatric conditions.

## Data Availability

The data are not publicly available due to their containing information that could compromise the privacy of research participants. Requests to access these datasets should be directed to JC, chenjx1110@163.com.
